# Comorbidities of scars in China: a national study based on hospitalized cases

**DOI:** 10.1093/burnst/tkab012

**Published:** 2021-06-10

**Authors:** Weishi Kong, Yongqiang Xiao, Baoli Wang, Zhe Zhu, Lunyang Hu, Hongtai Tang, Kangan Wang, He Fang, Ying Shi, Jianyan Long, Lanxia Gan, Haibo Wang, Yu Sun, Zhaofan Xia

**Affiliations:** 1 Department of Burn Surgery, the First Affiliated Hospital of Naval Medical University, Shanghai 200433, P.R.China; 2 Burn Institute of PLA, Research Unit of key techniques for treatment of burns and combined burns and trauma injury, Chinese Academy of Medical Sciences, Shanghai 200433, P.R.China; 3 Department of Burn and Plastic Surgery, The 970th Hospital of People's Liberation Army, Yantai, Shandong, 264000, China; 4 China Standard Medical Information Research Centre, Shenzhen 518000, P.R.China

**Keywords:** Scar, Epidemiology, Aetiology, Comorbidity, Chinese Hospital Quality Monitoring System, Hospitalization

## Abstract

**Background:**

Scar comorbidities seriously affect the physical and mental health of patients, but few studies have reported the exact epidemiological characteristics of scar comorbidities in China. This study aimed to investigate the prevalence of scar comorbidities in China.

**Methods:**

The data of 177,586 scar cases between 2013 and 2018 were obtained from the Hospital Quality Monitoring System based on the 10th edition of the International Classification of Diseases coding system. The total distribution of scar comorbidities and their relationship with age, aetiology and body regions were analysed.

**Results:**

Six comorbidities (contracture, malformation, ocular complications, adhesion, infection and others) were the main focus. In general, male patients outnumbered females and urban areas outnumbered rural areas. The proportion of contractures was the highest at 59,028 (33.24%). Students, workers and farmers made up the majority of the occupation. Han Chinese accounted for the majority of the ethnic. The highest proportion of scar contracture occurred at 1–1.9 years of age (58.97%), after which a significant downward trend was observed. However, starting from 50 years of age, ocular complications increased gradually and significantly, eventually reaching a peak of 34.49% in those aged >80 years. Scar contracture was the most common comorbidity according to aetiology, and the highest proportion was observed in patients who were scalded (29.33%). Contractures were also the most frequent comorbidity in hands (10.30%), lower limbs (6.97%), feet (6.80%) and upper limbs (6.02%). The mean and median hospitalization durations were 12.85 and 8 days, respectively.

**Conclusions:**

Contractures were the most common comorbidities, and different comorbidities tended to occur at different ages and with different causative factors.

Highlights

- We performed Big Data analyses on a medical database containing records of more than 80 million inpatients hospitalised in 1,064 tertiary hospitals in China.
- Scar patients suffer a lot from scar comorbidities and bear a heavy financial burden, but few reports have been made to illustrate the prevalence of scars in China or even in the world. This research conducted a cross-sectional study throughout China.
- This study analyzed in detail the scar comorbidities of 177586 scars caused by fire/flame, scalds, electrical burns, chemical burns, surgery, trauma, and acne, and covered 5 years of cases. This is a significant study and would contribute to the world literature of hypertrophic scarring.

## Background

Scarring is common in clinical practice, and there are numerous scar patients in China, the most populous country in the world [1]. Although scars are usually thicker than normal skin, they are more sensitive and fragile, which increases the risk of comorbidity formation, such as contraction, infection and malformation [2–5]. Patients with severe scar comorbidities often experience both aesthetic and psychosocial torment [6–8]. Patients’ daily life are also affected due to severe symptoms and complications, such as limited mobility, chronic pain and discomfort with their appearance. Scars can also cause serious damage to psychosocial health, especially in patients with facial scars [9]. The prevalence of anxiety and depression in scar patients is approximately 26.1 and 21.4%, respectively [10]. Many clinical treatments for scars exist, but very few are totally effective and satisfactory, and the costs may impose a financial burden on individuals and families. Therefore, the prevention and treatment of scar comorbidities is becoming the primary clinical focus.

Due to rapid economic development, an increasing number of skin injury and scar patients have been well-treated in China; however, many scar patients suffer from comorbidities every year [11,12]. At present, epidemiological surveys of scars are always limited to body region, age group, scar type or other specific characteristics in small scar populations from different parts of China [13–15]. Very few studies have examined the exact epidemiological characteristics of scar comorbidities in China, such as age, aetiology and region distribution. In this study, we aimed to conduct a cross-sectional study of the China National Survey of scar comorbidity cases reported in hospitals to evaluate the epidemiological characteristics and provide references for clinical prevention and treatment.

## Methods

To evaluate the characteristics of scars comorbidities in hospitalized patients, the Hospital Quality Monitoring System (HQMS) was used, a mandatory patient-level national database for hospital accreditation under the authority of the National Health and Family Planning Commission of the People’s Republic of China. The front-page information of all tertiary hospitals nationwide was recorded [16]. The International Statistical Classification of Diseases and Related Health Problems, 10th revision (ICD-10) provided the basis for the codes of scar cases, including the national ICD-10 edition, the Beijing edition and the clinical edition. All cases meeting the diagnostic codes were included. First, scar cases from 2013 to 2018 were identified from the HQMS using the ICD-10 codes, and then the comorbidity cases were screened from the scar cases according to the pre-defined ICD-10 comorbidity classification.

Ethical approval and consent were obtained for the data in this research from the Shanghai Changhai Hospital Ethics Committee (#CHEC2014–096). The patients’ clinical data can be used for research purposes.

In this study, some symptoms, sequelae and complications of scars were targeted, including contracture, malformation, ocular complications, adhesion, infection and others, which often coexist with severe scars in the clinic [17–19]. Other complications mainly included limb dysfunction and skin sensation. Collectively, symptoms, sequelae and complications are more appropriately referred to as scar comorbidities, because they are of different categories.

The trends of scar comorbidities, age, aetiologies and body regions were further studied. Due to the high proportion of comorbidities before the age of 20, the population was divided into the following age groups: <1 year, 1–1.9, 2–4.9, 5–9.9, 10–15.9, 16–19.9 years and every 10 years thereafter. Based on the data and previous studies, aetiologies were classified mainly as fire/flame burns, scalds, electrical burns, chemical burns, surgery, trauma and acne [20–23]. Body regions, including the eyes, ears, head and neck, upper limbs, hands, trunk, perineum, lower limbs and feet, were recorded [24]. The definitions and classification of all groups in this study were continuously revised according to data results by adjusting the ICD codes covered by each group, and a version with obvious data features and a more scientific classification was obtained.

SAS software (version 9.1, SAS Institute, Cary, NC) and R software (version 3.5.1, http://www.r-project.org/) were used for analyses. The numbers of cases were counted, rather than the number of patients.

## Results

### General characteristics of the study population

From 2013 to 2018, the comorbidities of all 177,586 hospital scar cases in the HQMS were included. The major comorbidities targeted in this study were divided into six categories: contracture (59,028, 33.24%), ocular complications (7604, 4.28%), malformation (6753, 3.80%), infection (3807, 2.14%), adhesion (3243, 1.83%) and other complications (168, 0.09%). The general characteristics of the study population are shown in Table 1. Overall, male scar patients outnumbered females; urban cases outnumbered rural cases in terms of infection, adhesion, ocular complications and other complications; while malformation and contracture were more frequent in rural cases. The most common occupations were students, workers and farmers (Table S1). In all ethnic groups, Han Chinese accounted for >89% of cases. The proportion of keloids was <2.41%.

**Table 1:** General characteristics of the study population^a^

**Comorbidities**		**Malformation**	**Infection**	**Contracture**	**Adhesion**	**Ocular complications**	**Other complications**
Sex, n(%)	Male	4300 (63.68)	2129 (55.92)	33 500 (56.75)	1912 (58.96)	4307 (56.64)	131 (77.98)
	Female	2443 (36.18)	1668 (43.81)	25 264 (42.8)	1308 (40.33)	3274 (43.06)	37 (22.02)
	Unknown	10 (0.15)	10 (0.26)	264 (0.45)	23 (0.71)	23 (0.3)	-
Urban or rural, n(%)	Urban	1215 (49.65)	1150 (62.13)	9137 (42.58)	620 (54.96)	2298 (61.97)	27 (71.05)
	Rural	1232 (50.35)	701 (37.87)	12 319 (57.42)	508 (45.04)	1410 (38.03)	11 (28.95)
Occupation, n(%)	Student	1159 (18.45)	348 (10.06)	10 277 (18.79)	336 (11.93)	498 (7.26)	26 (16.25)
	Worker	835 (13.29)	423 (12.23)	5256 (9.61)	367 (13.03)	494 (7.20)	38 (23.75)
	Farmer	655 (10.42)	556 (16.07)	4263 (7.80)	378 (13.42)	1531 (22.31)	21 (13.12)
	The others	3634 (57.84)	2133 (61.65)	34 888 (63.80)	1736 (61.63)	4339 (63.23)	75 (46.88)
Ethnic, n(%)	Han	4405 (89.15)	2746 (92.61)	41 659 (91.94)	2431 (94.12)	5828 (92.76)	139 (97.89)
	The others	536 (10.85)	219 (7.39)	3650 (8.06)	152 (5.88)	455 (7.24)	3 (2.11)
Keloid, n(%)	Yes	127 (0.58)	525 (2.41)	334 (1.53)	36 (0.17)	26 (0.12)	4 (0.02)
	No	21 650 (99.42)	21 252 (97.59)	21 443 (98.47)	21 741 (99.83)	21 751 (99.88)	21 773 (99.98)
Total, n(%)		6753 (3.80)	3807 (2.14)	59 028 (33.24)	3243 (1.83)	7604 (4.28)	168 (0.09)

^a^Cases with missing information were not included in the analysis: urban or rural, 49 975 (62.00%); occupation, 6337 (7.86%); ethnic, 18 380 (22.80%)

### Distributions of scar comorbidities in China from 2013 to 2018 by age

The proportions of comorbidities in different age groups are shown in Table 2. The highest proportion of scar contracture (58.97%) occurred at 1–1.9 years. After that, a significant downward trend was observed (4.23%). However, ocular complications exhibited a remarkable growth trend after 50 years of age, with 34.49% in those >80 years. The distribution of other comorbidities demonstrated no significant change across all ages, with proportions of ≤5.27%.

**Table 2:** The proportions of comorbidities in different age groups^a^

**Age (years)**	**Malformation n(%)**	**Infection n(%)**	**Contracture n(%)**	**Adhesion n(%)**	**Ocular complications n(%)**	**Other comorbidities n(%)**
<1	21 (1.91)	49 (4.45)	225 (20.45)	12 (1.09)	25 (2.27)	0 (0.00)
1–1.9	192 (4.97)	45 (1.16)	2279 (58.97)	66 (1.71)	18 (0.47)	5 (0.13)
2–4.9	810 (5.27)	190 (1.24)	8148 (53.01)	322 (2.09)	188 (1.22)	5 (0.03)
5–9.9	703 (3.89)	215 (1.19)	7463 (41.28)	301 (1.67)	388 (2.15)	13 (0.07)
10–15.9	649 (4.23)	197 (1.29)	6216 (40.55)	216 (1.41)	275 (1.79)	9 (0.06)
16–19.9	440 (4.02)	137 (1.25)	3552 (32.48)	146 (1.34)	264 (2.41)	6 (0.05)
20–29.9	1233 (3.61)	515 (1.51)	9732 (28.51)	551 (1.61)	839 (2.46)	43 (0.13)
30–39.9	934 (3.71)	523 (2.08)	9133 (36.24)	472 (1.87)	727 (2.88)	19 (0.08)
40–49.9	1043 (4.39)	732 (3.08)	6961 (29.28)	559 (2.35)	1073 (4.51)	39 (0.16)
50–59.9	543 (3.60)	539 (3.57)	3710 (24.61)	359 (2.38)	888 (5.89)	15 (0.10)
60–69.9	140 (1.61)	410 (4.72)	1183 (13.62)	148 (1.70)	1144 (13.17)	13 (0.15)
70–79.9	34 (0.80)	194 (4.59)	310 (7.33)	67 (1.58)	1162 (27.47)	1 (0.02)
80+	9 (0.56)	58 (3.60)	68 (4.23)	19 (1.18)	555 (34.49)	0 (0.00)

^a^Cases with missing age were not included in the analysis: 116 (0.14%)

### Distributions of scar comorbidities in China from 2013 to 2018 by aetiology

**Figure 1.**
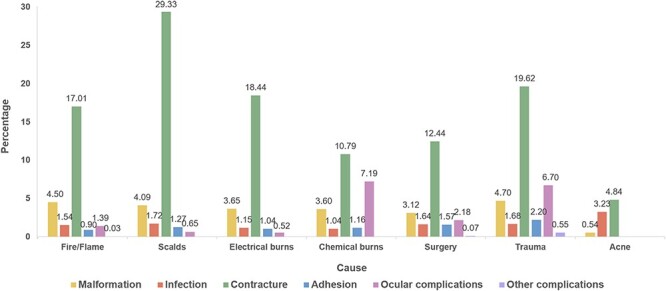
The proportions of different comorbidities of different causes

As shown in Figure 1, the aetiologies of scars were divided into seven main categories, including fire/flame burns, scalds, electrical burns, chemical burns, surgery, trauma and acne. Contracture had the highest proportion of all scar aetiologies, up to 29.33% in scald burns, followed by malformation and infection. Ocular complications were categorized into two groups: chemical burns (7.19%) and trauma (6.70%). There were only three main comorbidities of acne scars: contracture, infection and malformation, which is consistent with its clinical characteristics [17–19].

The proportions of scar contracture and ocular complications of different causes among ages are shown in Table 3, and the others are shown in Table S2, see online supplementary material. The proportions of contracture caused by fire/flame burns, scalds and trauma tended to decrease with age; higher proportions were mainly concentrated in age groups <16 years, with the highest proportions in those aged 1–1.9 years (78.57%, 61.11% and 53.33%, respectively). Ocular complications caused by surgery and trauma were the opposite, with the highest proportions in those aged 70–79.9 years (18.18 and 14.93%, respectively).

**Table 3:** The proportions of comorbidities of different causes^a^

**Age** **(years)**	**Contracture, n(%)**			**Ocular complications, n(%)**	
	**Fire/flame**	**Scalds**	**Trauma**	**Surgery**	**Trauma**
<1	1 (14.29)	8 (53.33)	6 (30.00)	—	—
1–1.9	22 (78.57)	121 (61.11)	8 (53.33)	0 (0.00)	1 (6.67)
2–4.9	98 (63.64)	354 (50.64)	88 (46.32)	4 (1.69)	5 (2.63)
5–9.9	148 (34.66)	301 (27.39)	97 (28.70)	7 (0.58)	10 (2.96)
10–15.9	89 (23.54)	179 (28.96)	64 (19.81)	9 (0.97)	16 (4.95)
16–19.9	48 (15.53)	57 (22.01)	50 (17.86)	7 (1.08)	17 (6.07)
20–29.9	196 (12.27)	88 (12.14)	173 (15.57)	41 (1.65)	76 (6.84)
30–39.9	164 (14.49)	42 (16.94)	166 (17.36)	46 (2.88)	69 (7.22)
40–49.9	249 (13.73)	48 (21.62)	193 (17.56)	44 (2.33)	79 (7.19)
50–59.9	126 (15.37)	24 (31.17)	183 (27.56)	18 (1.92)	53 (7.98)
60–69.9	36 (12.16)	5 (29.41)	39 (9.95)	23 (4.67)	24 (6.12)
70–79.9	12 (48.00)	0 (0.00)	7 (10.45)	22 (18.18)	10 (14.93)
80+	1 (16.67)	0 (0.00)	3 (10.71)	11 (17.46)	3 (10.71)
Total	1190 (17.02)	1227 (29.34)	1077 (19.64)	232 (2.18)	363 (6.62)

^a^Cases with missing age were not included in the analysis: flame, 1 (0.01%); scalds, 1 (0.02%); chemical burns, 5 (0.58%); surgery, 1 (0.01%); trauma, 7 (0.13%)

### Distributions of scar comorbidities in China from 2013 to 2018 by body regions

**Figure 2.**
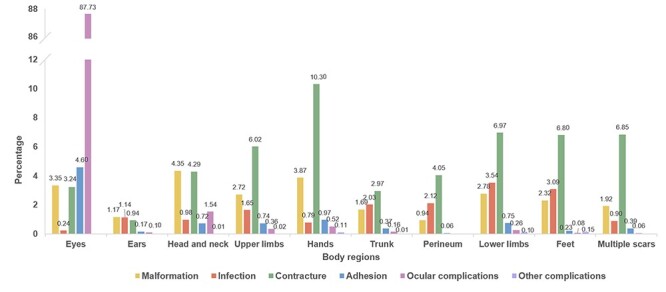
The proportions of comorbidities of different body regions

The proportions of body regions affected are shown in Figure 2 and Table 4. The proportion of ocular complications in the eyes group was very high (87.73%). Adhesion was significantly higher in the eye (4.60%) than in other groups. The proportions of the eyes (3.35%), head and neck (4.35%) and hands (3.87%) were high in the malformation group, which were also functional groups. The top three body regions with infection were the lower limbs (3.54%), feet (3.09%) and perineum (2.12%). The proportions of contracture were higher in the hands (10.30%), feet (6.80%), upper limbs (6.02%) and lower limbs (6.97%) than in other groups.

**Table 4:** The proportions of comorbidities of different body regions^a^

**Comorbidity**	**Eyes**	**Ears**	**Hands and neck**	**Upper limbs**	**Head**	**Trunk**	**Perineum**	**Lower limbs**	**Feet**	**Multiple scars**
Malformation, n(%)	268 (3.35)	35 (1.17)	800 (4.35)	150 (2.72)	172 (3.87)	133 (1.69)	15 (0.94)	107 (2.78)	30 (2.32)	89 (1.92)
Infection, n(%)	19 (0.24)	34 (1.14)	180 (0.98)	91 (1.65)	35 (0.79)	160 (2.03)	34 (2.12)	136 (3.54)	40 (3.09)	42 (0.90)
Contracture, n(%)	259 (3.24)	28 (0.94)	789 (4.29)	332 (6.02)	457 (10.30)	234 (2.97)	65 (4.05)	268 (6.97)	88 (6.80)	318 (6.85)
Adhesion, n(%)	368 (4.60)	5 (0.17)	132 (0.72)	41 (0.74)	43 (0.97)	29 (0.37)	1 (0.06)	29 (0.75)	3 (0.23)	18 (0.39)
Ocular complications, n(%)	7023 (87.73)	3 (0.10)	283 (1.54)	20 (0.36)	23 (0.52)	13 (0.16)	0 (0.00)	10 (0.26)	1 (0.08)	3 (0.06)
Other comorbidities, n(%)	0 (0.00)	0 (0.00)	2 (0.01)	1 (0.02)	5 (0.11)	1 (0.01)	0 (0.00)	4 (0.10)	2 (0.15)	0 (0.00)

^a^Cases with missing body region were not included in the analysis: 67 132 (83.29%)

### Hospitalization durations

The mean hospitalization durations for different comorbidities shown in Table 5 varied between 8.99 ± 14.63 and 19.49 ± 30.59 days, which means that the data is largely discrete, and the median was between 6 (Q1–Q3: 3–10) days and 13 (Q1–Q3: 7–22) days. The hospitalization duration of the infection group was the highest, and the mean and median of the ocular complications group were the lowest.

**Table 5:** Mean and median of hospitalization durations^a^

**Comorbidities**	**Hospitalization durations (days)**	
	**Mean ± (SD)**	**Median (Q1–Q3)**
Malformation	17.21 (33.79)	12 (7–18)
Infection	19.49 (30.59)	13 (7–22)
Contracture	15.50 (23.95)	11 (6–18)
Adhesion	14.29 (22.30)	10 (6–17)
Ocular complications	8.99 (14.63)	6 (3–10)
Other comorbidities	16.24 (19.40)	8 (6–20)
Overall	12.85 (30.72)	8 (5–15)

^a^Cases with missing information were not included in the analysis: hospitalization duration, 428 (0.53%)

Q1 refers to the number in the 25th percentile of a set of data arranged in sequence. Q3 refers to the number at the 75% position in a set of data arranged in sequence

## Discussion

Scars and their comorbidities might affect the physical and mental health of patients, and treatment fees also bring a huge economic burden to the country and society [25]. Therefore, the prevention and treatment of scar comorbidities is the focus of clinical attention.

Few reports have been made to illustrate the prevalence of scars and comorbidities in developing countries, most of which are limited by many factors, such as specific regions. There have been no explicit reports on the overall prevalence of scars in China. Here, an informative national database of most tertiary hospitals was used to determine the extent of scarring in hospitalized patients and to establish a unique data set to explore the demographic characteristics of scars and comorbidities in China.

The proportion of scar comorbidities was closely related to age. Different age groups have different mobility and self-protection capabilities, and therefore face various injury factors. Children and the elderly are more vulnerable to risk factors in daily life because of a lack of self-protection [26,27]. In addition, age may affect the demands and effectiveness of rehabilitation.

Children and juveniles are easily exposed to fire/flame, scalds and trauma in homes and schools due to their high mobility and low self-protection awareness. Other studies have reported that the incidence of hypertrophic scarring from burns is as high as 32–72%, and these scars are prone to develop into contracture [7]. Young age and deeper skin colour (pigmentation) are also important risk factors [28]. Scars tend to proliferate in children, and their lack of cooperation in rehabilitation training leads to a higher proportion of contracture [29]. In China, due to the increased attention paid to families and society, the rate of hospital visits is high, and these contribute to youths’ high contracture ratios.

Elderly individuals have poor physical function and face a high probability of encountering traumas such as car accidents: e.g. there is a high incidence of eye trauma in those aged 41–50 years and in those aged 51–60 years in south-central China [30]. Ocular complications are caused by scar symptoms, such as contractures of the scar around the eyes, eyelid ectropion and trichiasis. The anatomical part is analysed separately because ocular complications are complicated and often require specialized ophthalmology treatment, and they have certain characteristics in our data results. Studies have shown that the proportion of complications in ophthalmic surgery increases with advanced age [31] and may be associated with poor eye function and skin repair ability in elderly patients [32]. Furthermore, the anatomy of eyes is complex, the visual function is exquisite, and the scope is small, which means more difficult surgery and more challenging recovery, therefore the lack of development of rehabilitation training and recovery means may be one of the reasons for the high proportion.

The proportion of scar comorbidities is also closely related to its location. As functional regions are highly mobile and have a higher possibility of injury, there is a higher possibility of hospitalization and greater rehabilitation requirements in more functional regions. However, there is a lack of relevant research into the relationship between body regions and scar comorbidities worldwide [33]. In this report, contracture was more common in the hands, feet, and upper and lower limbs. Although the hands only account for 3–5% of the body’s surface area, they are often injured because of easy contact with risk factors, such as fire, and hands are often used to protect other parts of the body [14,34]. Second, the hand is an important functional part, where severe burns and independent injuries not involving other parts of the body often occur, especially in low- and middle-income countries with handicraft-leading manufacturing [35], leading to a higher proportion of scars. Due to their high functional requirements and thicker sole tissue, surgical reconstruction is poor and postoperative recovery is difficult, resulting in a high probability of comorbidities. In addition, in China, the use of rubber hot-water bottles to keep warm in winter is popular, especially for the feet when sleeping. Ruptured hot-water bottles can lead to scalds, and long contact between the bottle and skin can also lead to foot burns [11]. In short, the proportions of comorbidities with scarred functional parts such as the eyes, hands, feet, and upper and lower limbs were high, which may be due to their high structural and functional requirements, which also increases the rate of hospital visits and the possibility of hospitalization.

The longest hospitalization duration was in the infection group and the lowest was in the ocular complications group. Wound healing and scarring are closely associated with inflammation [36]. Scar infection is often delayed and difficult to cure, requiring long-term drug changes and care, especially for burn infections [37]. Clinical medicine, surgery and other treatment methods for eye scars are relatively mature, and the repair effect is good [38].

From this study, we conclude that clinicians should focus more on contracture and ocular complications in the elderly patients with scar comorbidities. Different causes of injury may affect the occurrence of complications. Functional parts, such as hands, feet and limbs, have a higher proportions of comorbidities than other parts. The duration of hospitalization for patients with scar comorbidities was concentrated around 10 days. These findings can be used as a reference for clinicians and scar patients. Scar comorbidities can harm physical and mental health and reduce the quality of life, and more attention should be paid to them clinically.

However, this study has certain limitations. First, HQMS only involves inpatients in China’s tertiary care facilities, while patients in military medical institutions, other hospitals, emergency rooms and clinics were not included. Second, the current research is essentially cross-sectional; therefore, a causal relationship cannot be inferred from our results. In addition, factors such as selectivity bias and irregular filling of medical records may also affect the research results.

## Conclusions

Scar comorbidity reports in China suggest that Chinese burn clinicians can learn from experience in other countries to educate high-risk people about safety and pay more attention to the health-related quality of life of burn patients after injury. In developing countries, scar comorbidities can be prevented through workplace and home safety measures to prevent injury, knowledge of self-rescue and timely medical treatment after injury, correct treatment of injury, and early prevention and treatment of scars after injury. This report is the first comprehensive epidemiological study and analysis of scar comorbidities in China, and it can be used for the prevention and treatment of these comorbidities.

## Supplementary Material

Table_S1_tkab012

Table_S2_tkab012

## Data Availability

The dataset used during the current study is available from the corresponding author upon reasonable request.
