# Supplementary material for: Comorbidities of scars in China: a national study based on hospitalized cases
Source: Burns Trauma. 2021 Jun 10;9:tkab012. doi: 10.1093/burnst/tkab012 (PMC8240520; doi:10.1093/burnst/tkab012)
Supplement: Table_S1_tkab012 [file table_s1_tkab012.docx]

**Table S1. Other occupations of comorbidities**

| **Occupation**  **n(%)** | **Malformation**  **n(%)** | **Infection**  **n(%)** | **Contracture**  **n(%)** | **Adhesion**  **n(%)** | **Ocular**  **Complications**  **n(%)** | **Other**  **Complications**  **n(%)** |
| --- | --- | --- | --- | --- | --- | --- |
| **National**  **public servant** | 106(1.69) | 42(1.21) | 559(1.02) | 71(2.52) | 112(1.63) | 4(2.5) |
| **Technical personnel** | 169(2.69) | 86(2.49) | 1,389(2.54) | 131(4.65) | 83(1.21) | 1(0.62) |
| **Staff** | 300(4.77) | 172(4.97) | 2,060(3.77) | 112(3.98) | 274(3.99) | 24(15) |
| **Enterprise manager** | 9(0.14) | 7(0.2) | 98(0.18) | 12(0.43) | 11(0.16) | - |
| **Free lances** | 133(2.12) | 59(1.71) | 785(1.44) | 50(1.77) | 152(2.22) | 4(2.5) |
| **Self-employed** | 71(1.13) | 38(1.1) | 376(0.69) | 34(1.21) | 72(1.05) | 2(1.25) |
| **Unemployed** | 630(10.03) | 366(10.58) | 5,877(10.75) | 295(10.47) | 611(8.9) | 9(5.62) |
| **Retirees** | 68(1.08) | 220(6.36) | 533(0.97) | 90(3.19) | 697(10.16) | 2(1.25) |
| **Others** | 2,148(34.19) | 1,143(33.03) | 23,211(42.45) | 941(33.4) | 2,327(33.91) | 29(18.12) |
