# Supplementary material for: Comorbidities of scars in China: a national study based on hospitalized cases
Source: Burns Trauma. 2021 Jun 10;9:tkab012. doi: 10.1093/burnst/tkab012 (PMC8240520; doi:10.1093/burnst/tkab012)
Supplement: Table_S2_tkab012 [file table_s2_tkab012.docx]

**Table S2. The proportions of comorbidities of different causes**

| **Cause** | **Age** | **Fire/flame** | **Scalds**  **n(%)** | **Electrical**  **Burns**  **n(%)** | **Chemical**  **Burns**  **n(%)** | **Surgical**  **n(%)** | **Trauma**  **n(%)** | **Acne**  **n(%)** |
| --- | --- | --- | --- | --- | --- | --- | --- | --- |
| **Malformation** | <1 | 1 (14.29) | 1 (6.67) | -- | -- | 1 (2.86) | 1 (5.00) | 0 (0.00) |
|  | 1-1.9 | 3 (10.71) | 12 (6.06) | -- | 0 (0.00) | 1 (4.55) | 0 (0.00) | -- |
|  | 2-4.9 | 15 (9.74) | 42 (6.01) | 1 (5.56) | 1 (11.11) | 5 (2.11) | 12 (6.32) | -- |
|  | 5-9.9 | 23 (5.39) | 45 (4.09) | 0 (0.00) | 0 (0.00) | 31 (2.59) | 30 (8.88) | -- |
|  | 10-15.9 | 15 (3.97) | 18 (2.91) | 2 (7.41) | 1 (2.94) | 25 (2.70) | 24 (7.43) | 0 (0.00) |
|  | 16-19.9 | 14 (4.53) | 4 (1.54) | 3 (12.50) | 2 (9.52) | 24 (3.71) | 12 (4.29) | 0 (0.00) |
|  | 20-29.9 | 73 (4.57) | 23 (3.17) | 9 (4.23) | 3 (2.63) | 84 (3.37) | 78 (7.02) | 0 (0.00) |
|  | 30-39.9 | 69 (6.10) | 9 (3.63) | 12 (10.62) | 8 (2.75) | 74 (4.62) | 42 (4.39) | 1 (4.35) |
|  | 40-49.9 | 73 (4.02) | 14 (6.31) | 4 (1.40) | 9 (3.07) | 72 (3.81) | 41 (3.73) | 0 (0.00) |
|  | 50-59.9 | 22 (2.68) | 3 (3.90) | 4 (1.80) | 2 (4.26) | 12 (1.28) | 13 (1.96) | 0 (0.00) |
|  | 60-69.9 | 5 (1.69) | 0 (0.00) | 0 (0.00) | 5 (41.67) | 3 (0.61) | 5 (1.28) | 0 (0.00) |
|  | 70-79.9 | 2 (8.00) | 0 (0.00) | -- | -- | 1 (0.83) | 0 (0.00) | -- |
|  | 80+ | -- | -- | -- | -- | -- | -- | -- |
|  | Total | 315 (4.50) | 171 (4.09) | 35 (3.65) | 31 (3.62) | 333 (3.12) | 258 (4.71) | 1 (0.54) |
| **Infection** | <1 | 0 (0.00) | 0 (0.00) | -- | -- | 2 (5.71) | 0 (0.00) | 0 (0.00) |
|  | 1-1.9 | 0 (0.00) | 3 (1.52) | -- | 0 (0.00) | 0 (0.00) | 0 (0.00) | -- |
|  | 2-4.9 | 2 (1.30) | 13 (1.86) | 0 (0.00) | 0 (0.00) | 2 (0.84) | 4 (2.11) | -- |
|  | 5-9.9 | 3 (0.70) | 21 (1.91) | 0 (0.00) | 2 (5.88) | 26 (2.17) | 5 (1.48) | -- |
|  | 10-15.9 | 7 (1.85) | 3 (0.49) | 0 (0.00) | 0 (0.00) | 15 (1.62) | 3 (0.93) | 0 (0.00) |
|  | 16-19.9 | 2 (0.65) | 1 (0.39) | 0 (0.00) | 0 (0.00) | 11 (1.70) | 3 (1.07) | 3 (5.88) |
|  | 20-29.9 | 20 (1.25) | 11 (1.52) | 6 (2.82) | 2 (1.75) | 25 (1.00) | 13 (1.17) | 3 (3.49) |
|  | 30-39.9 | 19 (1.68) | 5 (2.02) | 3 (2.65) | 3 (1.03) | 31 (1.94) | 17 (1.78) | 0 (0.00) |
|  | 40-49.9 | 38 (2.09) | 9 (4.05) | 0 (0.00) | 1 (0.34) | 29 (1.53) | 24 (2.18) | 0 (0.00) |
|  | 50-59.9 | 13 (1.59) | 2 (2.60) | 0 (0.00) | 1 (2.13) | 19 (2.02) | 17 (2.56) | 0 (0.00) |
|  | 60-69.9 | 4 (1.35) | 2 (11.76) | 2 (5.88) | 0 (0.00) | 8 (1.62) | 6 (1.53) | 0 (0.00) |
|  | 70-79.9 | 0 (0.00) | 2 (50.00) | -- | -- | 6 (4.96) | 0 (0.00) | -- |
|  | 80+ | 0 (0.00) | 0 (0.00) | -- | 0 (0.00) | 1 (1.59) | 0 (0.00) | -- |
|  | Total | 108 (1.54) | 72 (1.72) | 11 (1.15) | 9 (1.05) | 175 (1.64) | 92 (1.68) | 6 (3.23) |
| **Contracture** | <1 | 1 (14.29) | 8 (53.33) | -- | -- | 3 (8.57) | 6 (30.00) | 0 (0.00) |
|  | 1-1.9 | 22 (78.57) | 121 (61.11) | -- | 0 (0.00) | 5 (22.73) | 8 (53.33) | -- |
|  | 2-4.9 | 98 (63.64) | 354 (50.64) | 11 (61.11) | 7 (77.78) | 68 (28.69) | 88 (46.32) | -- |
|  | 5-9.9 | 148 (34.66) | 301 (27.39) | 9 (37.50) | 11 (32.35) | 163 (13.62) | 97 (28.70) | -- |
|  | 10-15.9 | 89 (23.54) | 179 (28.96) | 14 (51.85) | 8 (23.53) | 107 (11.56) | 64 (19.81) | 0 (0.00) |
|  | 16-19.9 | 48 (15.53) | 57 (22.01) | 11 (45.83) | 2 (9.52) | 73 (11.28) | 50 (17.86) | 3 (5.88) |
|  | 20-29.9 | 196 (12.27) | 88 (12.14) | 49 (23.00) | 15 (13.16) | 265 (10.64) | 173 (15.57) | 5 (5.81) |
|  | 30-39.9 | 164 (14.49) | 42 (16.94) | 35 (30.97) | 18 (6.19) | 209 (13.06) | 166 (17.36) | 1 (4.35) |
|  | 40-49.9 | 249 (13.73) | 48 (21.62) | 28 (9.82) | 14 (4.78) | 271 (14.32) | 193 (17.56) | 0 (0.00) |
|  | 50-59.9 | 126 (15.37) | 24 (31.17) | 17 (7.66) | 16 (34.04) | 116 (12.35) | 183 (27.56) | 0 (0.00) |
|  | 60-69.9 | 36 (12.16) | 5 (29.41) | 3 (8.82) | 1 (8.33) | 33 (6.69) | 39 (9.95) | 0 (0.00) |
|  | 70-79.9 | 12 (48.00) | 0 (0.00) | -- | -- | 11 (9.09) | 7 (10.45) | -- |
|  | 80+ | 1 (16.67) | 0 (0.00) | -- | 1 (100.00) | 1 (1.59) | 3 (10.71) | -- |
|  | Total | 1,190 (17.02) | 1,227 (29.34) | 177 (18.44) | 93 (10.85) | 1,325 (12.43) | 1,077 (19.64) | 9 (4.84) |
| **Adhesion** | <1 | -- | -- | -- | -- | -- | -- | -- |
|  | 1-1.9 | 0 (0.00) | 4 (2.02) | -- | 0 (0.00) | 0 (0.00) | 0 (0.00) | -- |
|  | 2-4.9 | 5 (3.25) | 18 (2.58) | 0 (0.00) | 0 (0.00) | 6 (2.53) | 1 (0.53) | -- |
|  | 5-9.9 | 6 (1.41) | 13 (1.18) | 0 (0.00) | 0 (0.00) | 10 (0.84) | 7 (2.07) | -- |
|  | 10-15.9 | 11 (2.91) | 3 (0.49) | 0 (0.00) | 0 (0.00) | 10 (1.08) | 5 (1.55) | 0 (0.00) |
|  | 16-19.9 | 2 (0.65) | 2 (0.77) | 1 (4.17) | 1 (4.76) | 8 (1.24) | 4 (1.43) | 0 (0.00) |
|  | 20-29.9 | 10 (0.63) | 7 (0.97) | 3 (1.41) | 2 (1.75) | 29 (1.16) | 30 (2.70) | 0 (0.00) |
|  | 30-39.9 | 11 (0.97) | 3 (1.21) | 0 (0.00) | 2 (0.69) | 25 (1.56) | 26 (2.72) | 0 (0.00) |
|  | 40-49.9 | 8 (0.44) | 2 (0.90) | 4 (1.40) | 4 (1.37) | 39 (2.06) | 18 (1.64) | 0 (0.00) |
|  | 50-59.9 | 7 (0.85) | 1 (1.30) | 2 (0.90) | 1 (2.13) | 19 (2.02) | 21 (3.16) | 0 (0.00) |
|  | 60-69.9 | 2 (0.68) | 0 (0.00) | 0 (0.00) | 0 (0.00) | 14 (2.84) | 5 (1.28) | 0 (0.00) |
|  | 70-79.9 | 1 (4.00) | 0 (0.00) | -- | -- | 6 (4.96) | 3 (4.48) | -- |
|  | 80+ | 0 (0.00) | 0 (0.00) | -- | 0 (0.00) | 1 (1.59) | 0 (0.00) | -- |
|  | Total | 63 (0.90) | 53 (1.27) | 10 (1.04) | 10 (1.17) | 167 (1.57) | 120 (2.19) | 0 (0.00) |
| **Ocular**  **complications** | <1 | -- | -- | -- | -- | -- | -- | -- |
|  | 1-1.9 | 0 (0.00) | 0 (0.00) | -- | 0 (0.00) | 0 (0.00) | 1 (6.67) | -- |
|  | 2-4.9 | 4 (2.60) | 0 (0.00) | 0 (0.00) | 0 (0.00) | 4 (1.69) | 5 (2.63) | -- |
|  | 5-9.9 | 3 (0.70) | 3 (0.27) | 0 (0.00) | 1 (2.94) | 7 (0.58) | 10 (2.96) | -- |
|  | 10-15.9 | 3 (0.79) | 1 (0.16) | 0 (0.00) | 0 (0.00) | 9 (0.97) | 16 (4.95) | 0 (0.00) |
|  | 16-19.9 | 4 (1.29) | 0 (0.00) | 0 (0.00) | 3 (14.29) | 7 (1.08) | 17 (6.07) | 0 (0.00) |
|  | 20-29.9 | 18 (1.13) | 6 (0.83) | 0 (0.00) | 6 (5.26) | 41 (1.65) | 76 (6.84) | 0 (0.00) |
|  | 30-39.9 | 22 (1.94) | 9 (3.63) | 3 (2.65) | 9 (3.09) | 46 (2.88) | 69 (7.22) | 0 (0.00) |
|  | 40-49.9 | 29 (1.60) | 1 (0.45) | 1 (0.35) | 26 (8.87) | 44 (2.33) | 79 (7.19) | 0 (0.00) |
|  | 50-59.9 | 6 (0.73) | 5 (6.49) | 1 (0.45) | 10 (21.28) | 18 (1.92) | 53 (7.98) | 0 (0.00) |
|  | 60-69.9 | 7 (2.36) | 1 (5.88) | 0 (0.00) | 2 (16.67) | 23 (4.67) | 24 (6.12) | 0 (0.00) |
|  | 70-79.9 | 0 (0.00) | 0 (0.00) | -- | -- | 22 (18.18) | 10 (14.93) | -- |
|  | 80+ | 0 (0.00) | 0 (0.00) | -- | 0 (0.00) | 11 (17.46) | 3 (10.71) | -- |
|  | Total | 96 (1.37) | 26 (0.62) | 5 (0.52) | 57 (6.65) | 232 (2.18) | 363 (6.62) | 0 (0.00) |
| **Other**  **Comorbidities** | <1 | -- | -- | -- | -- | -- | -- | -- |
|  | 1-1.9 | -- | -- | -- | -- | -- | -- | -- |
|  | 2-4.9 | -- | -- | -- | -- | -- | -- | -- |
|  | 5-9.9 | 1 (0.23) | 0 (0.00) | 0 (0.00) | 0 (0.00) | 0 (0.00) | 0 (0.00) | -- |
|  | 10-15.9 | 0 (0.00) | 0 (0.00) | 0 (0.00) | 0 (0.00) | 2 (0.22) | 0 (0.00) | 0 (0.00) |
|  | 16-19.9 | -- | -- | -- | -- | -- | -- | -- |
|  | 20-29.9 | 0 (0.00) | 0 (0.00) | 0 (0.00) | 0 (0.00) | 1 (0.04) | 9 (0.81) | 0 (0.00) |
|  | 30-39.9 | 0 (0.00) | 0 (0.00) | 0 (0.00) | 0 (0.00) | 0 (0.00) | 4 (0.42) | 0 (0.00) |
|  | 40-49.9 | 1 (0.06) | 0 (0.00) | 0 (0.00) | 0 (0.00) | 3 (0.16) | 11 (1.00) | 0 (0.00) |
|  | 50-59.9 | 0 (0.00) | 0 (0.00) | 0 (0.00) | 0 (0.00) | 1 (0.11) | 2 (0.30) | 0 (0.00) |
|  | 60-69.9 | 0 (0.00) | 0 (0.00) | 0 (0.00) | 0 (0.00) | 0 (0.00) | 3 (0.77) | 0 (0.00) |
|  | 70-79.9 | 0 (0.00) | 0 (0.00) | -- | -- | 0 (0.00) | 1 (1.49) | -- |
|  | 80+ | -- | -- | -- | -- | -- | -- | -- |
|  | Total | 2 (0.03) | 0 (0.00) | 0 (0.00) | 0 (0.00) | 7 (0.07) | 30 (0.55) | 0 (0.00) |

Cases with missing information were not included in the analysis. Fire/flame: 1(0.01%), Scalds: 1(0.02%), Chemical burns: 5(0.58%), Surgical: 1(0.01%), Trauma: 7(0.13%).
